# Sentinel node detection in N0 cancer of the pharynx and larynx

**DOI:** 10.1038/sj.bjc.6600445

**Published:** 2002-09-23

**Authors:** J A Werner, A-A Dünne, A Ramaswamy, B J Folz, B M Lippert, R Moll, Th Behr

**Affiliations:** Department of Otolaryngology, Head and Neck Surgery, Philipps-University of Marburg, Deutschhausstr. 3, 35037 Marburg, Germany; Department of Pathology, Philipps-University of Marburg, Deutschhausstr. 3, 35037 Marburg, Germany; Department of Clinical Nuclear Medicine, Philipps-University of Marburg, Deutschhausstr. 3, 35037 Marburg, Germany

**Keywords:** sentinel node, N0 neck, squamous cell carcinoma, larynx, pharynx, occult metastases

## Abstract

Neck lymph node status is the most important factor for prognosis in head and neck squamous cell carcinoma. Sentinel node detection reliably predicts the lymph node status in melanoma and breast cancer patients. This study evaluates the predictive value of sentinel node detection in 50 patients suffering from pharyngeal and laryngeal carcinomas with a N0 neck as assessed by ultrasound imaging. Following 99m-Technetium nanocolloid injection in the perimeter of the tumour intraoperative sentinel node detection was performed during lymph node dissection. Postoperatively the histological results of the sentinel nodes were compared with the excised neck dissection specimen. Identification of sentinel nodes was successful in all 50 patients with a sensitivity of 89%. In eight cases the sentinel node showed nodal disease (pN1). In 41 patients the sentinel node was tumour negative reflecting the correct neck lymph node status (pN0). We observed one false-negative result. In this case the sentinel node was free of tumour, whereas a neighbouring lymph node contained a lymph node metastasis (pN1). Although we have shown, that skipping of nodal basins can occur, this technique still reliably identifies the sentinel nodes of patients with squamous cell carcinoma of the pharynx and larynx. Future studies must show, if sentinel node detection is suitable to limit the extent of lymph node dissection in clinically N0 necks of patients suffering from pharyngeal and laryngeal squamous cell carcinoma.

*British Journal of Cancer* (2002) **87**, 711–715. doi:10.1038/sj.bjc.6600445 www.bjcancer.com

© 2002 Cancer Research UK

## 

Paralell to other tumour entities there were intensified efforts within the last two decades, which are still discussed controversially, to limit the extent of lymph node dissection in the clinically staged N0 situations also for head and neck cancer patients. The aim of reducing a potential excess of surgical therapy for the patient is currently achieved quite successfully in other tumour entities by applying the so-called sentinel node (SN) concept (Lingam *et al*, 1997; de Cicco *et al*, 2000; Lantzsch *et al*, 2001). The extensive investigations on large patient cohorts in breast cancer and malignant melanoma are opposed by a comparative paucity of experience with the SN concept for SCC of the upper aerodigestive tract (Pitman *et al*, 1998; Shoaib *et al*, 1999, 2001; Alex *et al*, 2000; Chiesa *et al*, 2000; Colnot *et al*, 2001; Stoeckli *et al*, 2001). Most of these articles predominantely deal with oral cancer. Contrary to this, it was the aim of the present study to analyse the role of the SN procedure in patients with pharyngeal and laryngeal carcinoma, which are the most frequent cancers of the upper aerodigestive tract. Primary criterion for inclusion was the N0 neck as staged by ultrasound scanning. Further inclusion criterion was the feasibility of a transoral exposure of the tumour. Adequate injection of the tracer substance especially in the caudal margin of the tumour. Due to inadequate exposure of the tumour three patients (2x larynx, 1x hypopharynx) had to be excluded from the study.

## PATIENTS AND METHODS

This study was approved by an ethics committee and written informed consent was obtained from the patients at the beginning of therapy.

### Patients

In 50 previously untreated patients (6 female, 44 male; age range: 33–79 years, median: 63 years) suffering from squamous cell carcinomas of the oropharynx (*n*=33), the larynx (*n*=14) and the hypopharynx (*n*=3) an intraoperative SN detection was performed. B-mode-ultrasound assessed a N0 neck in all 50 patients. Lymph nodes >1 cm in diameter, lymph nodes with a spherical appearance or diffuse borders to the surrounding soft tissue were defined as criteria for malignancy in neck sonography. These cases required fine needle aspiration cytology and if they were preoperatively classified as N1-neck these cases were not enrolled in this study. Further details on the location and TNM-classification can be withdrawn from Table 1

**Table 1:**
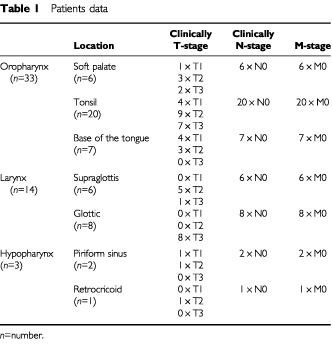
Patients data

.

### Methods

At the beginning of the surgery (tumour resection and neck dissection) the application of a total amount of 1.2 mCi Tc99m-nanocolloid, which was dissolved in 0.2–0.35 ml normal saline, was performed by intraoperative injections. This amount was chosen under the consideration of the special anatomical challenges in the lymphatic system of the upper aerodigestive tract, which is characterised by regional variation, but high density of lymphatics. An excessive amount of tracer substance leads to drainage into several neighbouring lymph node levels due to an unphysiological increase of the interstitial pressure. Before this background, it has to be pointed out, that more than 100 years ago Mascagni could show, that lymphatic fluids usually pass up to eight lymph nodes until they enter the blood vessels.

The tracer substance, which had been aspirated into a 1 ml syringe (Plastipak®, Becton Dickinson, Madrid, Spain) was injected into four spots at the perimeter of the tumour under microscopic control. In cases of easily accessible SCCs of the oropharynx a hypodermic 24 Gauge needle of 25 mm length (Microlance3®, Becton Dickinson, Drogheda, Ireland) was used. Laryngeal and hypopharyngeal carcinomas were exposed via rigid endoscopy in general anaesthesia. The tracer substance was then injected under microscopic control into the perimeter of the tumour, using a 23 Gauge needle of 80 mm length (Sterican®, B Braun, Melsungen, Germany), ensuring that no considerable amounts of the tracer were spilled.

Based on the results of previous studies (Werner *et al*, 1999), which will be summarised briefly in the Discussion, preoperative lymphoscintigraphy was not performed in this investigation. Blue dye injection was also not used to identify the first draining lymph nodes.

Using a 14-mm diameter straight collimated probe (Navigator, Gamma Guidance System, Autosuture, Toenisvorst, Germany) the intraoperative identification of the sentinel nodes was performed within a maximum delay of 6 h (time range 2–6 h). This time span which may seem extended is based on the fact that in certain cases a bilateral neck dissection (*n*=20) with identification of radiolabelled lymph nodes on both sides of the neck had to be performed. Indications for bilateral neck dissection were carcinomas, which extended to the midline or trespassed it. With one exception all supraglottic carcinomas (5/6) were also treated by bilateral neck dissection. The exception to the rule was a patient suffering from a small T2 carcinoma of the supraglottis which was located on the free edge of the epiglottis with a wide clear margin to the midline. The extent of neck dissection depended on the size and likelihood of occult metastatic spread.

Subsequent intraoperative readings with the gamma probe were performed to identify the sentinel nodes. Readings were usually performed after elevation of the cutaneous flaps. In the following, the lymph node containing tissue of the predominant metastatic region (usually level II or III) was mobilized, until the operation specimen could be rotated carefully. It was necessary to increase the distance between neck dissection specimen and primary injection site of the tracer substance to reduce the influence of radioactive scattering on identification of the SN with the gamma probe. The identified hot nodes were excised and extra-corporal control readings were performed. Due to the fact that especially carcinomas of the upper aerodigestive tract may initially drain into more than one lymph node, the three hottest nodes, containing more than 10 times the background level of radioactivity, were defined as sentinel nodes (SN1-3). Unilateral (*n*=30) or bilateral (*n*=20) neck dissection was then performed. Following uni- or bilateral neck dissection, the primary tumour was excised either by conventional surgery (*n*=3) or laser surgery (*n*=47), depending on the size and location of the lesion. In the remaining eight patients, in whom the carcinoma had been removed transorally by laser microsurgery, the operation had been performed directly prior to neck dissection, which facilitated intraoperative SN detection. The main cause for improved detection of the sentinel nodes was the elimination of scattered radiation from the peritumoural tissue. When performing this type of procedure, which is currently favoured by us, a time space of 15–20 min passes between tracer injection and laser microsurgical resection of the primary.

In this study the sentinel nodes as well as each lymph node of the neck dissection specimen were divided along the equatorial plane into two halves, which then were embedded in paraffin. Serial sections at intervals of 1 mm were sliced resulting in complete work-up of the entire lymph node. The sections were then stained with H&E. One section per lymph node was stained with PAS, and another section was stained immunhistochemically for ceratin using the pan-cytokeratin antibody MNF 116 (Chemicon, Hofheim, Germany).

## RESULTS

In all 50 patients one or more sentinel nodes could be detected intraoperatively. A total number of 90 sentinel nodes could be identified. Sixteen out of ninety sentinel nodes were found on the contralateral side in carcinomas, which were either situated in the midline or expanded over the midline (Table 2

**Table 2:**
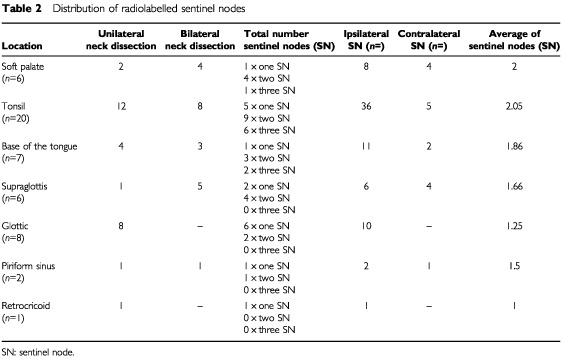
Distribution of radiolabelled sentinel nodes

). Corresponding to the varying density of the lymphatics of the upper aerodigestive tract, the median number of identified hot nodes depended on the location of the primary tumour. When calculating median values it became evident, that carcinomas of the oropharynx and the supraglottis had two sentinel nodes, whereas glottic carcinomas usually had only one first draining lymph node.

During pathohistologic examination a total of 2538 lymph nodes were analysed. On an average 36 lymph nodes (range 15–67) were examined histologically per neck dissection specimen (*n*=70).

Forty-one out of 50 patients had tumour free sentinel nodes reflecting the neck regional lymph node status (pN0), while in seven patients a solitary macrometastasis (H&E) and in one patient a micrometastasis (MNF 116) was proven during histopathological examination (pN1(mi)). In the remaining patient (T2 oropharyngeal carcinoma) a pitfall occurred (Table 3

**Table 3:**
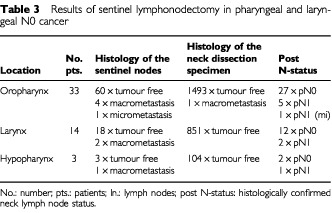
Results of sentinel lymphonodectomy in pharyngeal and laryngeal N0 cancer

). The intraoperatively identified SN was shown to be tumour free during histopathological investigation. However, a directly neighbouring lymph node histologically proved to contain a metastasis of 0.65 cm in diameter with perinodal spread (pN1). This lymph node had not been suspicious on macroscopic examination. Intraoperative and extracorporal readings of the dissected neck dissection specimen had shown no tracer accumulation within this lymph node. Thus there were nine patients with nodal disease and the procedure correctly identified eight of these (Table 4

**Table 4:**
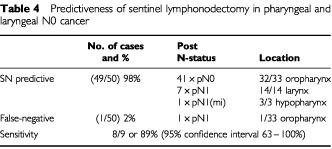
Predictiveness of sentinel lymphonodectomy in pharyngeal and laryngeal N0 cancer

). Therefore, the sensitivity of sentinel node detection in this study was 8/9 or 89% (95% confidence interval 63–100%).

In this clinically N0-neck patient group the rate of occult nodal disease was found to be 18%. The rate of micrometastases (<2 mm) with a size between 0.5–1 mm within the neck dissection specimen was 87.5% (7/8) in cases of histologically proven macrometastases within the sentinel nodes (*n*=8) and 0% (0/41) in cases of tumour free sentinel nodes (*n*=41). The number of isolated tumour cells was 21.9% (9/41) in cases of tumourfree sentinel nodes. In the patient with a false negative sentinel node the neck dissection specimens (pN1) contained one further micrometastases.

## DISCUSSION

The controversy on the extent of surgical therapy of the clinically unsuspicious lymphatic drainage region of a malignant tumour occupies all surgical specialties. With regard to this background SN detection proved to be an accurate technique in melanoma and breast cancer patients, in order to predict lymph node status (Ferwerda *et al*, 2000; Gennari *et al*, 2000; Veronesi *et al*, 2001). Corresponding to previously reported results in SN detection in easily accessible SCC of the upper aerodigestive tract like oral carcinoma (van den Brekel *et al*, 1999; Nieuwenhuis *et al*, 2000; Margolin *et al*, 2001; Zitsch *et al*, 2000), the SN concept may also be of significance in pharyngeal and laryngeal cancer, which so far have rarely been the topic of targeted investigations.

In contrast to the procedure in breast cancer we abstain from performing a preoperative dynamic lymphoscintigraphy as well as from additional injection of blue dye. In previous investigations (Werner *et al*, 1999) it could be shown, that transcutaneous readings in the area of the deep jugular lymph node level II–IV allow no exact identification of the sentinel nodes. Furthermore the depiction of lymphatic drainage in dynamic scintigraphy was tightly bound to the quality of the injection technique. Interferences through a poor exposure of the tumour, sudden movements of the patient or gagging become evident and obscured the depiction of the lymphatic drainage into the representative main draining level of a primary tumour. Due to the fact, that intraoperative approach renders good exposure and precise injections because of less interference by the patient, we exclusively prefer the intraoperative injection technique even in carcinomas of the soft palate or tonsil. In carcinomas of the deeper parts of the pharynx and larynx the intraoperative injection technique usually is mandatory.

We abstained from blue dye injection for several reasons. An accidental damage to the lymphatic duct may lead to extravasation of blue dye, which might not only cause impaired overall view of the surgical field, but could also lead to delayed wound healing (Borgstein *et al*, 1998). Furthermore it has to be mentioned, that as early as in the year of 1985 anaphylactic reactions after subcutaneous injections of blue dye had been reported (Longnecker *et al*, 1985), which may be evident in 2% of the investigated cases, as reflected by the current literature (Cimmino *et al*, 2001). With regard to these complications and the resulting potential danger of accidental damage to functionally relevant structures the additional application of blue dye does not seem to be justified, especially when considering that the scintigraphy method gives good results.

Based on the detailed analyses of the lymphatics of the human upper aerodigestive tract (Werner, 1995a,b) it can be said, that the direction of the lymphatic drainage of this area is relatively constant. This was also shown for the main direction of the metastatic spread in carcinomas of the head and neck (Lindberg, 1972). Exact knowledge of these metastatic patterns allow a targeted mobilization of this tissue, which contains the respective lymph nodes in the predominant region of metastatic spread of a primary. Its rotation renders the intraoperative identification of the radiolabelled sentinel nodes with less interference of the scattered radiation of the primary injection side. The recently developed strategy to remove the primary tumour laser surgically prior to performing the neck dissection has proven to be beneficial with this regard. The potential disadvantage of radiation scattering from the primary injection site, which may impede the identification of the sentinel node, may thus be eliminated to the greatest possible extent.

The results, which can be achieved with this method are promising. Sentinel nodes free of disease did reflect the correct stage of the regional nodal status (pN0) – with respect to the known direct influence of the prognosis – in 41 of 50 investigated pharyngeal and laryngeal cancer patients. At this point it should not be omitted, that, despite a rate of 0% for micrometastases, the rate of isolated tumour cells in the remaining neck dissection specimen was found to be 21.9%. But it has to be pointed out, that this finding was determined throughout an advanced histopathological examination, which does not seem to be feasible for the clinical routine due to high costs and expenditure of labour. The status of such an advanced work-up of the neck dissection specimen can not be estimated at present. We would like to agree with Hermanek (1999), who proposed that the introduction of an advanced examination of lymph nodes regarding isolated or disseminated tumour cells first of all requires standardization of the procedure and description of the clinical relevance. It seems to be out of the question, that an additional reference to isolated tumour cells (suffix: i) or micrometastasis (suffix: mi) would be desirable in a histopathologic report, as it was recommended by the UICC 1999. However, as long as the independent prognostic significance of isolated tumour cells is still unclear, it is not going to be considered in the further discussion.

The detection of solitary macrometastasis (pN1) in seven patients and of a micrometastasis (pN1(mi)) in one patient with clinically staged N0 neck seemed to be very interesting. The detailed investigation of each lymph node of the neck dissection specimen revealed in 7/8 patients 1–2 further micrometastases within the lymph nodes of the neck dissection specimen. Among 41 patients without macrometastases were 0/41 patients suffering from micrometastases and 9/41 patients in whom isolated tumour cells could be detected. Thus, the rate of occult metastatic spread in the presented investigation was 18% for nodal disease, which is in accordance with the literature for SCC of the head and neck (Hosal *et al*, 2000). With regard to these results radiolabelled SN detection showed a sensitivity of 89%. These findings suggest that the SN method renders detection of lymphogenic metastatic spread also in patients with carcinomas of the pharynx and larynx.

This statement is of relevance especially for T3 glottic carcinomas. Glottic carcinomas, which are sometimes staged by the pathologist as T2 tumours, or rarely even as T3 tumours because of fixed vocal cords. This is an excellent example for the necessity of interdisciplinary communication in finding the correct diagnosis. Resection of the previously mentioned T3 glottic tumours may be possible for especially trained surgeons by laser micro surgery in selected cases. Provided that good exposure of the tumour is given as inclusion criterion for laser micro surgery, a targeted peritumoural injection of the tracer substance poses no difficult problem for laser surgically trained surgeons. It should not be omitted to point out, that the tracer injection may be difficult at the caudal margin.

If this technique does not seem to be feasible, we would consider the procedure risky, if the caudal margin cannot be injected transtumourally under endoscopic control with the required reliability. This could be shown in three patients (2x larynx, 1x hypopharynx) which we not included in the present study for obvious reasons. For these situations we actually developed an injection via a butterfly cannula (Winged infusion set, 23G, 0.65×20 mm, Braun, Melsungen, Germany), which can be inserted in an oblique angle.

Some surgeons perform a selective neck dissection in T3 glottic carcinomas because of the important influence of lymphogenic metastatic spread on prognosis of patients with carcinomas of the head and neck, although the likelihood of occult lymphogenic metastatic spread in T3 glottic carcinoma is only estimated to be 10–15% (Sinard *et al*, 1996). The critical analysis of further multicentric trials reporting on SN detection in T3 glottic carcinomas may lead to a more precise estimation of the risk of occult metastatic spread and thus eventually to a more limited surgical approach in this special indication.

Despite of the encouraging results we would like to point out, that perinodal spread may be a reason for non-accumulation of a tracer substance, which has been reported previously (Borgstein *et al*, 1998). The phenomenon of perinodal spread, which is usually seen in advanced metastatic spread, may also be evident in smaller metastases (Croce *et al*, 1992; Coatesworth and Maclennan, 2002), as could also be shown in one of our patients. The head and neck surgeon has to be aware of this fact, when performing and interpreting the results of SN detection in head and neck cancer.

In summary, although we have shown, that skipping of nodal basin can occur, this technique still reliably identifies the sentinel nodes in pharyngeal and laryngeal cancer. Usually this applies to laryngeal and supraglottic T1-T2 carcinomas as well as T3 glottic carcinoma. The limiting factor of the feasibility of this technique seems to be the reliable caudal margin of the tumour.

In conclusion it has to be assumed, that SN detection in carcinomas of the upper aerodigestive tract is first of all a diagnostic procedure. Through a targeted histopathological examination in serial sections the radiolabelled sentinel nodes may help to increase the diagnostic reliability of a limited neck dissection. This may help in decision making, whether more radical surgery is required, whether postoperative radio(chemo)therapy is necessary or whether a wait and see policy is justified.

The radioguided dissection of the 2–3 hottest nodes and the surrounding tissue may be a further step in reduction of radicality in surgery of the clinically staged N0 neck. However, the value of such an approach has to be examined in multicentric trials. As future perspective it may be possible to direct the efforts on a development of an endoscopic neck dissection (Carreno *et al*, 1999; Dulguerov *et al*, 2001), which would then be performed as a very limited form of neck dissection. Preliminary own experiences show that further pursuance of this aim seems to be justified.
